# Pay-for-performance as a cost-effective implementation strategy: results from a cluster randomized trial

**DOI:** 10.1186/s13012-018-0774-1

**Published:** 2018-07-04

**Authors:** Bryan R. Garner, Aung K. Lwin, Gail K. Strickler, Brooke D. Hunter, Donald S. Shepard

**Affiliations:** 1 RTI International, P. O. Box 12194, Research Triangle Park, Raleigh, NC 27709-2194 USA; 2 Schneider Institutes for Health Policy, The Heller School, MS035, Brandeis University, Waltham, MA USA; 3 Minnesota Department of Human Services, Saint Paul, MN USA

**Keywords:** Implementation research, Cost-effectiveness, Substance use, Adolescent

## Abstract

**Background:**

Pay-for-performance (P4P) has been recommended as a promising strategy to improve implementation of high-quality care. This study examined the incremental cost-effectiveness of a P4P strategy found to be highly effective in improving the implementation and effectiveness of the Adolescent Community Reinforcement Approach (A-CRA), an evidence-based treatment (EBT) for adolescent substance use disorders (SUDs).

**Methods:**

Building on a $30 million national initiative to implement A-CRA in SUD treatment settings, urn randomization was used to assign 29 organizations and their 105 therapists and 1173 patients to one of two conditions (implementation-as-usual (IAU) control condition or IAU+P4P experimental condition). It was not possible to blind organizations, therapists, or all research staff to condition assignment. All treatment organizations and their therapists received a multifaceted implementation strategy. In addition to those IAU strategies, therapists in the IAU+P4P condition received US $50 for each month that they demonstrated competence in treatment delivery (A-CRA competence) and US $200 for each patient who received a specified number of treatment procedures and sessions found to be associated with significantly improved patient outcomes (target A-CRA). Incremental cost-effectiveness ratios (ICERs), which represent the difference between the two conditions in average cost per treatment organization divided by the corresponding average difference in effectiveness per organization, and quality-adjusted life years (QALYs) were the primary outcomes.

**Results:**

At trial completion, 15 organizations were randomized to the IAU condition and 14 organizations were randomized to the IAU+P4P condition. Data from all 29 organizations were analyzed. Cluster-level analyses suggested the P4P strategy led to significantly higher average total costs compared to the IAU control condition, yet this average increase of 5% resulted in a 116% increase in the average number of months therapists demonstrated competence in treatment delivery (ICER = $333), a 325% increase in the average number of patients who received the targeted dosage of treatment (ICER = $453), and a 325% increase in the number of days of abstinence per patient in treatment (ICER = $8.134). Further supporting P4P as a cost-effective implementation strategy, the cost per QALY was only $8681 (95% confidence interval $1191–$16,171).

**Conclusion:**

This study provides experimental evidence supporting P4P as a cost-effective implementation strategy.

**Trial registration:**

NCT01016704.

## Background

Illicit drug use has been estimated to cost the USA an approximately $200 billion annually as a result of direct and indirect costs associated with crime, health, and productivity [1], which surpasses the estimated costs for other chronic health issues like diabetes [2] and obesity [3]. Although a number of evidence-based treatments (EBTs) to address substance use disorders (SUDs) have been developed [4], implementation of EBTs for SUDs within practice settings has been limited [5, 6]. This gap has lowered the return-on-investment of both research-related dollars spent developing EBTs and service-related dollars spent providing treatment to individuals and families that are in need of SUD services.

Beyond the relatively low levels of EBT implementation found within the SUD treatment field [5, 6], EBT implementation has been identified as a significant challenge for other areas of health [7–10]. In 2007, as part of a broad effort to improve the quality of care delivered within the USA (including greater implementation of EBTs), the Institute of Medicine recommended pay-for-performance (P4P) as a promising strategy to improve implementation of high-quality care [11]. This recommendation, combined with the limited empirical research evidence supporting P4P as a method to improve quality of care, motivated an experimental test of the effectiveness and cost-effectiveness of P4P as a strategy to improve the implementation and effectiveness of the Adolescent Community Reinforcement Approach (A-CRA)—an EBT shown to be effective and cost-effective in treating SUDs for adolescents [12–18]. The rationale for using a cluster randomized trial design was that primary interest was to examine P4P as an organizational-level strategy, as well as that validity threats are possible from the randomization of patients within therapists (e.g., contamination) or of therapists within treatment organizations (e.g., compensatory rivalry and resentful demoralization).

Main effectiveness findings suggested P4P had a direct impact on improving the two primary measures of implementation (one measured at the therapist level and one measured at the patient level), as well as an indirect impact on patient substance use [19]. That is, relative to therapists in the implementation-as-usual (IAU) condition [20], therapists in the IAU+P4P condition were significantly more likely to demonstrate monthly A-CRA competence (event rate ratio = 2.24). Additionally, relative to patients treated in the IAU condition, patients treated in the IAU+P4P condition were significantly more likely to receive an empirically supported level of A-CRA treatment exposure (target A-CRA; odds ratio = 5.19), which was positively associated with post-treatment recovery status (odds ratio = 1.91). According to a recent systematic review of quality improvement, implementation, and dissemination strategies to improve mental health care for children and adolescents [21], Garner and colleagues [19] findings provided “The strongest evidence in the review” for their key question (i.e., What is the effectiveness of quality improvement, implementation, and dissemination strategies).

Consistent with the original study protocol [22], recommendations for evaluating the cost-effectiveness of P4P [23, 24], and the importance of SUD research addressing cost issues [25], the primary purpose of the current study was to report on the incremental cost-effectiveness of P4P as a discrete implementation strategy. We hypothesized that relative to the IAU condition, the IAU+P4P condition would have significantly higher total implementation costs (hypothesis 1), significantly lower incremental cost-effectiveness ratio (ICER) per therapist month of A-CRA competence (hypothesis 2), significantly lower ICER per patients receiving target A-CRA (hypothesis 3), and significantly lower ICER per day of abstinence at follow-up (hypothesis 4). Additionally, we hypothesized (hypothesis 5) that the cost per quality-adjusted life year (QALY) would be less than the USA per capita gross domestic product, which is the standard recommended by the World Health Organization for an intervention to be highly cost-effective [26].

## Methods

All study procedures were conducted under Institutional Review Board approval. The study protocol paper for this cluster randomized trial was published in *Implementation Science* in 2010 [19, 27, 28]. The current article was written in accordance with the 2012 CONSORT guidelines for cluster randomized trials [29], which were published 1 month after publication of the effectiveness paper [19]. Additionally, in appreciation of the importance of implementation strategy specification, Table 1 specifies each of the discrete implementation strategies included as part of the IAU condition (see A–J of Table 1) and the P4P strategy (see K of Table 1) included as an adjunct to the IAU condition. Table 1 was developed to be consistent with the list of discrete implementation strategies compiled by Powell and colleagues [30], as well as with Proctor and colleagues [31] recommendations regarding implementation strategy specification.

**Table 1:** Name, definition, and operationalization of each discrete implementation strategy

Discrete implementation strategies: defining characteristic according to Proctor and colleagues [31]	Operational definition of key dimensions for each discrete implementation strategy				
	Actor(s)	Actions(s)	Target(s) of the action	Temporality/dose	Justification
A. Centralized technical assistance: Develop and use a system to deliver technical assistance focused on implementation issues.	The A-CRA developer team contracted to help implement A-CRA as part of the SAMHSA/CSAT-funded implementation initiative.	Technical assistance contract awarded to Chestnut Health System’s EBT coordinating center by SAMHSA/CSAT.	Therapists selected to learn to implement A-CRA as part of the SAMHSA/CSAT-funded implementation initiative.	Ongoing throughout the SAMHSA/CSAT-funded implementation initiative.	[57–60]
B. Develop educational materials: Develop and format guidelines, manuals, toolkits, and other supporting materials in ways that make it easier for stakeholders to learn about the innovation and for clinicians to learn how to deliver the clinical innovation.	The A-CRA developer team contracted to help implement A-CRA as part of the SAMHSA/CSAT-funded implementation initiative.	The A-CRA protocol manual [61], which provides information and knowledge about how the MIBI is intended to be implemented.	Therapists selected to learn to implement A-CRA as part of the SAMHSA/CSAT-funded implementation initiative.	Developed prior to the start of the SAMHSA/CSAT-funded implementation initiative.	[73, 74]
C. Develop and organize quality monitoring system: Develop and organize systems and procedures that monitor clinical processes and/or outcomes for quality assurance and improvement.	The A-CRA developer team contracted to help implement A-CRA as part of the SAMHSA/CSAT-funded implementation initiative.	A Web-based tool (EBTx.org) that enables secure and efficient sharing of A-CRA session information and audio recordings.	Therapists selected to learn to implement A-CRA as part of the SAMHSA/CSAT-funded implementation initiative.	Developed prior to the start of the SAMHSA/CSAT-funded implementation initiative.	[20, 75, 76]
D. Develop tools for quality monitoring: Develop, test, and introduce quality-monitoring tools with inputs (e.g., measures) specific to the innovation being implemented.	The A-CRA developer team contracted to help implement A-CRA as part of the SAMHSA/CSAT-funded implementation initiative.	The A-CRA coding manual [62], which enables rating of A-CRA fidelity.	Therapists selected to learn to implement A-CRA as part of the SAMHSA/CSAT-funded implementation initiative.	Developed prior to the start of the SAMHSA/CSAT-funded implementation initiative.	[63–65]
E. Distribute educational materials: Distribute educational materials (e.g., manuals) in-person, by mail, and/or electronically.	The A-CRA developer team contracted to help implement A-CRA as part of the SAMHSA/CSAT-funded implementation initiative.	Distribute copies of the A-CRA manual [61] to therapists.	Therapists selected to learn to implement A-CRA as part of the SAMHSA/CSAT-funded implementation initiative.	Distributed approximately one month prior to the SAMHSA/CSAT-funded implementation initiative’s in-person training workshop.	[73, 74, 77]
F. Conduct educational meetings: Hold meetings targeted toward providers, administrators, other organizational stakeholders, and community, patient or consumer, and family stakeholders to teach them about the clinical innovation.	The A-CRA developer team contracted to help implement A-CRA as part of the SAMHSA/CSAT-funded implementation initiative.	In-person workshop training that enables direct interaction between the actors (A-CRA developers) and targeted users (therapists).	Therapists selected to learn to implement A-CRA as part of the SAMHSA/CSAT-funded implementation initiative.	In-person 3.5 day training workshop at the beginning of the SAMHSA/CSAT-funded implementation initiative, with similar training workshops provided approximately every 6–12 months throughout the SAMHSA/CSAT-funded implementation initiative.	[57, 66, 78, 79]
G. Make training dynamic: Vary the information delivery methods to cater to different learning styles and work contexts and shape the training in the innovation to be interactive.	The A-CRA developer team contracted to help implement A-CRA as part of the SAMHSA/CSAT-funded implementation initiative.	Incorporate role plays that enable therapists to practice implementing A-CRA procedures.	Therapists selected to learn to implement A-CRA as part of the SAMHSA/CSAT-funded implementation initiative.	As possible throughout the SAMHSA/CSAT-funded implementation initiative.	[66–69]
H. Audit & provide feedback: Collect and summarize clinical performance data over a specified period, and give data to clinicians and administrators in the hopes of changing provider behavior.	The A-CRA developer team contracted to help implement A-CRA as part of the SAMHSA/CSAT-funded implementation initiative.	Generate and email feedback reports based on ratings of session audio recordings that were rated using the A-CRA coding manual [62].	Therapists selected to learn to implement A-CRA as part of the SAMHSA/CSAT-funded implementation initiative.	Approximately weekly prior to demonstrating A-CRA proficiency and then approximately monthly throughout the remainder of SAMHSA/CSAT-funded implementation initiative.	[78, 80–83]
I. Provide ongoing consultation: Provide clinicians with continued consultation with an expert in the clinical innovation.	The A-CRA developer team contracted to help implement A-CRA as part of the SAMHSA/CSAT-funded implementation initiative.	Individual coaching that enables direct contact between the actor (A-CRA developer) and a targeted user (therapist).	Therapists selected to learn to implement A-CRA as part of the SAMHSA/CSAT-funded implementation initiative.	Approximately weekly prior to demonstrating A-CRA proficiency and then approximately monthly throughout the remainder of SAMHSA/CSAT-funded implementation initiative.	[57, 78, 79]
J. Create a learning collaborative: Develop and use groups of providers or provider organizations that will implement the clinical innovation and develop ways to learn from one another to foster better implementation.	The A-CRA developer team contracted to help implement A-CRA as part of the SAMHSA/CSAT-funded implementation initiative.	Group coaching meetings that enable direct contact between the actor (A-CRA developer) and a group of targeted users (therapists).	Therapists selected to learn to implement A-CRA as part of the SAMHSA/CSAT-funded implementation initiative.	Monthly throughout the SAMHSA/CSAT-funded implementation initiative.	[70–72]
K. Use other payment schemes: Introduce payment approaches motivate the clinician to provide better service.	Our research team funded by NIAAA to test the incremental effectiveness and cost-effectiveness of P4P as an implementation strategy.	$50 for each month a therapist demonstrated competence in treatment delivery (A-CRA competence) and $200 for each patient who received at least the targeted number of treatment procedures and sessions (target A-CRA).	Therapists selected to learn to implement A-CRA as part of the SAMHSA/CSAT-funded implementation initiative and who work at organizations randomized to the IAU+P4P condition.	Monthly throughout the NIAAA-funded cluster randomized trial.	[11, 54]

### Study overview

Twenty-nine community-based treatment organizations (97% participation rate) were assigned via urn randomization to either the control IAU condition (*n* = 15) or the experimental IAU+P4P condition (*n* = 14). It was not possible to blind organizations, therapists, or all research staff to condition assignment. As part of a national EBT implementation initiative conducted between October 2006 and October 2010 by the Substance Abuse and Mental Health Services Administration’s Center for Substance Abuse Treatment (SAMHSA/CSAT) [4, 20], treatment organizations in both conditions received similar levels of funding to implement A-CRA. Additionally, funded by SAMHSA/CSAT as part of a separate contract to A-CRA model developers, treatment organizations in both conditions received a multifaceted implementation strategy to assist with their implementation of A-CRA (see A–J of Table 1). The level of standardization provided by the SAMHSA/CSAT initiative provided a unique opportunity to test the incremental effectiveness and cost-effectiveness of P4P as a discrete implementation strategy (see K of Table 1).

In September 2008, via funding from the National Institute on Alcohol Abuse and Alcoholism, Garner and colleagues initiated the reinforcing therapist performance (RTP) experiment [22]. With enrollment from November 17, 2008, through January 12, 2009, this cluster randomized experiment involved 29 treatment organizations, 105 therapists, and 1173 patients. As noted above, IAU for the current cluster randomized trial included a multifaceted implementation strategy (A–J of Table 1) [30]. In addition to the discrete implementation strategies included in the IAU condition, therapists who worked at one of the 14 treatment organizations randomized to the IAU+P4P condition and who provided voluntary written consent to participate in the study were offered the opportunity to earn monetary bonuses for achievement of two predefined treatment implementation performance measures (i.e., P4P; see K of Table 1). First, therapists could earn $200 for each of their patients who received target A-CRA (defined as at least 10 of 12 specific A-CRA procedures delivered within the first 14 weeks of treatment and in no fewer than 7 sessions). Several studies found target A-CRA to be significantly associated with decreased post-treatment substance use [13, 19, 22]. Second, to reinforce delivery of treatment procedures with high quality, therapists could earn $50 for each month that they demonstrated A-CRA competence (defined as competent delivery of all components of at least one A-CRA treatment procedure during the same treatment session). Demonstration of target A-CRA and A-CRA competence was determined by a trained rater based on review of session audio recordings. Overall, there was a 95% level of agreement between the study’s primary rater and monthly fidelity ratings conducted by a second rater blinded to study condition [19]. Each month, participating therapists working at one of the treatment organizations assigned to the IAU+P4P condition received emails regarding any monthly bonuses earned. Participating therapists received their bonuses monthly either through check or direct deposit. Conducted from the perspective of the healthcare system, the cost-effectiveness study focused on costs incurred by each participating treatment organization.

### Cost measures

There were three categories of system costs; all used 2010 US dollars. The first category, termed *Training & Coaching Costs*, included costs related to the training, coaching, certification, and fidelity monitoring of therapists delivering A-CRA treatment sessions to patients as part of the SAMHSA/CSAT initiative. These costs were estimated for each treatment organization by multiplying the treatment organization’s total number of participating therapists by $5717 (i.e., the overall average cost per therapist involved according to administrative training records).

The second category, termed *Treatment Costs*, included costs related to the delivery of A-CRA treatment sessions to patients. Treatment costs were calculated for each treatment organization by multiplying the treatment organization’s total number of treatment sessions by $125.26, which represents the inflation-adjusted (adjusted to 2010 dollars) average cost of a counseling hour as reported by the Alcohol and Drug Services Cost Study [32], a nationally representative study.

The third category, termed *P4P Costs*, included costs related to the monetary bonuses paid to therapists and costs associated with the administration of the P4P methods (e.g., staff time to determine achievement of the two performance measures and to issue monetary bonuses to therapists). Each treatment organization’s P4P cost associated with target A-CRA was computed by summing each treatment organization’s total amount of target A-CRA bonuses ($200 per target A-CRA) plus the product of the number of patients who were identified as having met target A-CRA eligibility criteria times $44.21 (i.e., the average fully loaded cost [including fringe and indirect costs] of 1.5 h of staff time to review each session’s audio recordings in order to verify each patient’s target A-CRA). Similarly, the treatment organization’s P4P cost associated with A-CRA competence was calculated by adding the amount of A-CRA competence bonuses ($50 per A-CRA competence) plus the product of the number of months therapists met the monthly eligibility criteria of recording at least 80% of their face-to-face A-CRA treatment sessions times $29.47 (i.e., the average fully loaded cost [including fringe and indirect costs] of 1.0 h of staff time to review a randomly selected session audio recording for A-CRA competence). Total P4P costs were computed for each treatment organization by summing all of their respective target A-CRA and A-CRA competence costs.

### Effectiveness measures

Effectiveness was assessed by implementation measures and health outcome measures. The implementation measures included the number of months therapists demonstrated A-CRA competence and the number of patients receiving target A-CRA. The first health outcome measure was the percent of days patients were abstinent from alcohol and other drugs at the 6-month follow-up. Data from both of the study conditions were combined to model the impact on patient abstinence as a function of receipt of target A-CRA. The sample was all patients (*n* = 600) with known outcome status at the 6-month follow-up assessment. Follow-up rates were similar (χ^2^ = 0.12, *p* = .73) in patients with (63%) and without target A-CRA (64%). Results indicated that patients who received target A-CRA reported a significantly greater percentage of days of abstinence from alcohol and drug use (coefficient = 0.153 [SE = 0.076], *p* < .05). Converting by 365 to years gave the added person-years of abstinence. The second outcome measure was the estimated number of quality-adjusted life years (QALYs) gained. Our QALY measure was calculated using the results of a study by Daley and colleagues (2005) [33], which extended the QALY measure to substance abuse. In the Daley et al. study, participants gained 0.19 QALYs over 1 year while their rates of abstinence improved by 55.6 percentage points, indicating that each percentage point improvement in abstinence (and concurrent improvements in other dimensions) over 1 year was associated with a gain of 0.00342 QALYs. This implies a disability weight per year for substance use overall of 0.342 (i.e., 0.00342/1%), with this weight measuring the amount lost from perfect health. Additionally, given that Daley and colleagues’ study represents only a single investigation of the overall QALY burden of substance abuse, we also calculated QALY using the results of a global study that estimated disability weights separately by substance and severity [34]. Among the five relevant substances, the median weights (across severity levels) ranged from 0.153 for cannabis to 0.516 for heroin, with the midpoint value (0.335) similar to the value derived using the Daley et al. study (0.342).

### Analysis plan

Consistent with standard economic theory [35], we assessed the cost-effectiveness of the P4P methods using incremental cost-effectiveness ratios (ICER). For each comparison, the ICER is the difference between the two arms in average yearly cost per treatment organization divided by the corresponding average difference in effectiveness per organization over 1 year. The comparison between the IAU condition and the IAU+P4P condition calculated the costs and impact of adding P4P onto the existing SAMHSA/CSAT program. We estimated the confidence interval (CI) of the cost per QALY by treating target A-CRA by treatment organization as a normal variable. The lower (most favorable) bound for the CI used the highest disability weight (0.516), where treatment would confer the greatest benefit. The upper (least favorable) bound for the CI used the lowest disability weight (0.153), where treatment would confer the least benefit. Finally, a preliminary cost-benefit analysis of IAU + P4P vs. IAU valued each QALY at the 2010 U.S. Gross National Income per capita of $48,950 [36].

## Results

**Fig. 1:**
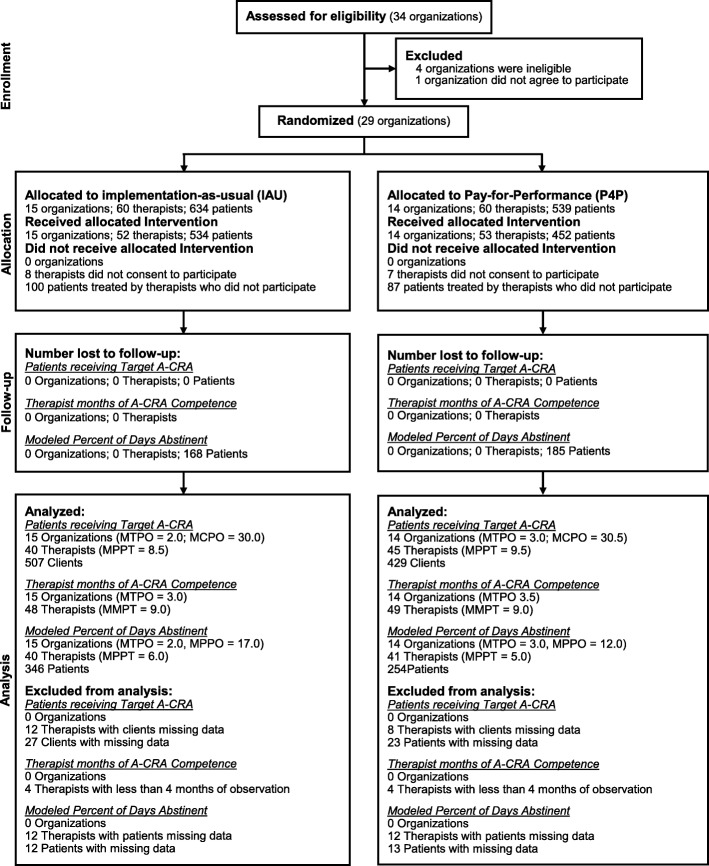
Flow of treatment organizations, therapists, and patients through the study. Notes: IAU indicates implementation-as-usual, P4P indicates pay-for-performance, A-CRA indicates adolescent community reinforcement approach, MTPO indicates median therapists per organization, MPPO indicates median patients per organization, MPPT indicates median patients per therapist, MMPT indicates median months per therapist

Figure 1 shows the flow of treatment organizations, therapists, and patients through the experiment. No adverse events were reported. Table 2 shows implementation outcomes and costs by condition. Relative to treatment organizations assigned to the IAU condition, treatment organizations assigned to the IAU+P4P condition had a significantly higher average number of months that therapists demonstrated A-CRA competence (IAU = 8.62; IAU+P4P = 18.64; *p* < 0.001; 116% increase) and a significantly higher average number of patients who received target A-CRA (IAU = 2.27; IAU+P4P = 9.64; *p* < 0.001; 325% increase).

**Table 2:** Implementation outcomes and costs per treatment organization by study condition

	IAU (*n* = 15)		IAU±P4P (*n* = 14)	
	Mean	SD	Mean	SD
Implementation outcomes				
A-CRA competence	8.62	7.58	18.64*	14.68
Target A-CRA	2.27	2.74	9.64*	11.31
Costs				
Training and coaching	$18,844	$8367	$23,483*	$11,472
Treatment	$44,073	$22,951	$39,838*	$15,051
P4P	NA	NA	$2935	$3103
A-CRA competence	NA	NA	$1076	$1013
Target A-CRA	NA	NA	$1859	$2273
Total	$62,917	$22,953	$66,256*	$25,006

Note: ^*^p < 0.001. IAU indicates implementation-as-usual, P4P indicates pay-for-performance, SD indicates standard deviation, A-CRA indicates adolescent community reinforcement approach, NA indicates not applicable

In terms of costs, the IAU+P4P condition had a significantly higher average Training & Coaching Cost (IAU = $18,844; IAU+P4P = $23,483; *p* < 0.001; 24.6% increase), but also a significantly lower average treatment cost (IAU = $44,073; IAU+P4P = $39,838; *p* < 0.001; 9.6% decrease) per organization. Additionally, when these costs were combined with P4P costs, which averaged $2935 (*SD* = $3103) for the treatment organizations assigned to the IAU+P4P condition, the total cost per organization was significantly higher for the IAU+P4P condition (IAU = $62,917; IAU+P4P = $66,256; *p* = .034; 5% increase).

Treatment costs represented the largest percentage of total cost for both conditions (70% for IAU and 58% for IAU+P4P), with Training & Coaching Costs representing the second largest percentage of total cost (30% for IAU and 34% for IAU+P4P). Within the IAU+P4P condition, the average P4P cost represented only 3% of the average total cost. The average amount of monetary bonuses paid per treatment organization was $1076 (*SD* = $1013) for A-CRA competence and $1859 (*SD* = $2273) for target A-CRA. Additionally, administration costs added 30% to the costs for the P4P incentives.

Table 3 presents the incremental costs, incremental effectiveness, and incremental cost-effectiveness ratios (ICERs). Relative to the IAU condition, the ICERs for therapist months of A-CRA competence, patients receiving target A-CRA, and days of abstinence per patient for the IAU+P4P condition were $333, $453, and $8.134, respectively. Table 3 also presents the incremental cost per QALY for patients, which was $8681 per QALY for the IAU+P4P vs. IAU. Using the range of disability weights from different substances [34], the ICER could range from $5754 (derived from the heroin disability) to $19,405 (derived from the cannabis disability). The value of $8681 is substantially below the 2010 US per capita GDP ($48,950) [36].

**Table 3:** Cost-effectiveness results

Contrast and indicator	Process measures per treatment organization		Outcome measures per patient	
	Therapist months of A-CRA competence	Patients receiving target A-CRA	Days of abstinence per patient	QALYs per patient
IAU±P4P versus IAU				
Incremental cost	$3338	$3338	$103	$103
Incremental effectiveness	10.02	7.37	12.72	0.0119
Incremental cost-effectiveness ratio*	$333	$453	$8.134	$8681

Note: *The ICER for cost per QALY ($8681) was calculated as $8.134 × 365/0.342 with a 95% confidence interval of $1191 to $16,171. IAU indicates implementation-as-usual, P4P indicates pay-for-performance, QALY indicates quality-adjusted life year, A-CRA indicates adolescent community reinforcement approach

## Discussion

P4P has been recommended as a promising strategy to improve the quality of care delivered within the United States [11]; however, empirical support for P4P based on rigorous experimental research has been limited to date [24, 37–41]. In an effort to address this gap, the current study examined the incremental cost-effectiveness of a P4P implementation strategy previously shown to be highly effective at improving the implementation and effectiveness of an EBT for adolescent SUDs [19].

Overall, study results suggest P4P can be a cost-effective implementation strategy. Supporting hypothesis 1, the addition of P4P led to a significantly higher average total cost relative to the IAU condition. Supporting hypotheses 2 through 4, despite the increase in average total cost, the IAU+P4P condition was found to be more cost-effective than the IAU condition regarding therapist months of A-CRA competence, as patient’s receipt of target A-CRA, and patient days of abstinence at follow-up. More specifically, the average increase of 5% in total costs resulted in a 116% increase in the average number of therapist months of A-CRA competence (ICER = 333), a 325% increase in the average number of patients who received target A-CRA (ICER = $453), and a 325% increase in the number of days of abstinence per patient in treatment (ICER = $8.134). According to a recent systematic review of economic evaluations of P4P in health care, only two full economic evaluations of P4P approaches had been evaluated through randomized trials [23]. The first study found that a 16% increase in cost raised the receipt of flu vaccination by 9% [42], while the second study found that a 971% increase in cost raised the number of referrals to a tobacco quitline by 236% [43]. Thus, results of the present study are unique as they represent the first known P4P experiment to find that the percentage increase in implementation outcomes was greater than the percentage increase in costs. Finally, supporting hypothesis 5, the cost per QALY ($8655) was more favorable (i.e., lower) than two benchmarks—the comparably calculated pooled average from Dennis and colleagues’ interventions ($11,641) [12] and the USA per capita GDP ($46,616) [44], the latter of which is the standard recommended by the World Health Organization for an intervention to be highly cost-effective [26].

Our use of the perspective of the healthcare system is increasingly recommended for its relevance to resource constraints of many decision makers [45]. An analysis from the societal perspective, the other option, would require complete societal costs, such as value of participants’ time and transportation expense. Given our study lacked direct information about such societal costs, our costs are underestimated. However, because therapists encouraged adolescents to engage in pro-social activities with low or no out-of-pocket costs to the adolescents (e.g., high-school event), as well as that the opportunity costs of the time of adolescents with substance abuse disorders are likely minimal, this omission is assumed to be minor.

Valuing each QALY at the per capita GDP of the USA based on a human capital approach [46], we conducted a preliminary benefit-cost analysis of IAU+P4P vs. IAU. It gave a ratio of 5.4 with a range, based on the disability weights for alternative substances, of 2.4 to 8.1, with all values above the cutoff of 1.0. These preliminary benefit-cost ratios are likely conservative, as we did not include possible reductions in crime nor the value that adolescents might place on participating in encouraged activities, such as watching high-school sports with their friends.

### Strengths and limitations

Important strengths of the current study include the use of a randomized design, independent verification of behaviors that resulted in receipt of P4P incentives, the inclusion of administration costs as part of the P4P costs, and links to QALY outcome measures. Important limitations of the current study, however, were reliance on patient self-report of substance use and substance-related problems, as well as the lack of biometric data (e.g., breathalyzer or urine test results).

### Implications and directions for future research

McCarty and colleagues [25] identified five priorities for policy research on treatment for substance use disorders, which included: (a) organization and delivery of care, (b) quality of care, (c) EBTs, (d) access to care, and (e) financing, costs, and value of care. With regard to “financing, costs, and value of care,” McCarty and colleagues [25], who specifically highlighted P4P as an emerging strategy, noted “policy makers are often more attentive to cost-effectiveness estimates than overall spending estimates.” Thus, one key implication of the current study is it provides estimates of the cost-effectiveness of P4P strategies. Given the limited number of studies that have experimentally tested the cost-effectiveness of P4P [23], in addition to having important implications for the SUD treatment field, current findings also may have important implications for other areas of healthcare that have struggled to identify effective and cost-effective models of P4P [24, 38, 39, 47, 48]. Nonetheless, additional estimates regarding the cost-effectiveness of P4P remain needed.

With regard to EBTs as an area of priority for policy research, McCarty and colleagues [25] noted the need to identify strategies to help facilitate use of EBTs. Thus, a second implication of the current study is it provides strong evidence that P4P can be a cost-effective strategy to help improve implementation of EBTs. This is important given identification of effective and cost-effective implementation strategies is an important topic for several areas of health [9]. Given the limited number of effective and cost-effective implementation strategies, additional research examining the effectiveness and cost-effectiveness of P4P as a discrete strategy or component of a multifaceted implementation strategy is warranted.

Finally, with regard to research on quality of care, McCarty and colleagues [25] noted “the most critical policy issues for research on quality of care pertain to the link between performance measures and evidence of enhanced treatment outcomes.” Thus, given the patient-level performance measure used in this study (target A-CRA) has been shown to be a significant predictor of patient outcomes [13, 19, 22], a third implication of the current study is that it supports focusing implementation strategies on improving evidenced-based measures of implementation [49], which are distinguished from other “implementation outcomes” that have not been shown to be associated with improved treatment outcomes (e.g., acceptability, appropriateness, feasibility).

## Conclusion

The present study provides experimental evidence supporting P4P as a cost-effective implementation strategy to improve implementation of EBTs for SUD treatment. The precise reasons why our P4P study had a large effect, in contrast to other large P4P studies that found P4P to have modest [50] to no impact [39], are not known; however, consistent with research highlighting the importance of the design elements of P4P [38, 48, 51–53], we believe at least three design issues were important. First, in contrast to P4P designs that focused on numerous performance measures, many of which had little room for improvement due to base rates above 90% [50], we designed the current study to focus on two clinically relevant performance measures that had considerable room for improvement due to their relatively low base rates [19, 22]. Second, rather than the P4P incentives being directed toward the organization, which is how most P4P studies to date have been designed [38], our study design directed the P4P incentives at the individual therapists. In addition to being consistent with our prior research that had used therapist-directed P4P incentives to improve client retention in SUD continuing care [54], this design choice is consistent with Van Herck and colleagues’ conclusion that “targeting the individual has generally better effects than not to do so” [48]. Third, in contrast to P4P designs where P4P incentives were provided on a relatively infrequent annual basis [39, 50], our study design provided P4P incentives on a relatively frequent monthly basis, which is consistent with key principles of operant conditioning [55, 56]. Future research is clearly needed, including the optimal bonus amounts and the extent to which effects are sustained. Our hope, however, is that the current P4P experiment will not only help inform and improve P4P research, but also will help inform and improve implementation research focused on identifying effective and cost-effective strategies for improving implementation of EBTs in practice settings.

## Abbreviations

A-CRA: Adolescent community reinforcement approach
CI: Confidence interval
CSAT: Center for Substance Abuse Treatment
EBT: Evidence-based treatment
IAU: Implementation-as-usual
ICERS: Incremental cost-effectiveness ratios
P4P: Pay-for-performance
QALY: Quality-adjusted life years
RTP: Reinforcing therapist performance
SAMHSA: Substance Abuse and Mental Health Services Administration
SUD: Substance use disorders
